# Fabrication of graded index single crystal in glass

**DOI:** 10.1038/srep44327

**Published:** 2017-03-13

**Authors:** Keith Veenhuizen, Sean McAnany, Daniel Nolan, Bruce Aitken, Volkmar Dierolf, Himanshu Jain

**Affiliations:** 1 Department of Physics, Lehigh University, 16 Memorial Drive East, Bethlehem, PA, 18015, USA; 2 Department of Materials Science and Engineering, 5 East Packer Avenue, Bethlehem, PA, 18015, USA; 3 Corning Incorporated, Corning, NY, 14831, USA

## Abstract

Lithium niobate crystals were grown in 3D through localized heating by femtosecond laser irradiation deep inside 35Li_2_O-35Nb_2_O_5_-30SiO_2_ glass. Laser scanning speed and power density were systematically varied to control the crystal growth process and determine the optimal conditions for the formation of single crystal lines. EBSD measurements showed that, in principle, single crystals can be grown to unlimited lengths using optimal parameters. We successfully tuned the parameters to a growth mode where nucleation and growth occur upon heating and ahead of the scanning laser focus. This growth mode eliminates the problem reported in previous works of non-uniform polycrystallinity because of a separate growth mode where crystallization occurs during cooling behind the scanning laser focus. To our knowledge, this is the first report of such a growth mode using a fs laser. The crystal cross-sections possessed a symmetric, smooth lattice misorientation with respect to the c-axis orientation in the center of the crystal. Calculations indicate the observed misorientation leads to a decrease in the refractive index of the crystal line from the center moving outwards, opening the possibility to produce within glass a graded refractive index single crystal (GRISC) optically active waveguide.

The growth of crystals in glass has been explored due to its potential application in optical data transmission. Several crystals have been fabricated through continuous wave^1^,^2^,^3^,^4^,^5^,^6^,^7^,^8^ (CW) and femtosecond^9^,^10^,^11^,^12^,^13^,^14^,^15^,^16^,^17^,^18^ (fs) laser induced heating of the host glass. While crystal growth induced via CW laser heating is limited to the glass surface, fs laser induced crystallization allows for crystal growth deep inside the glass. The use of fs lasers to alter the properties of a host material has opened a compelling opportunity for expanding integrated optics into dense, 3D geometries. Besides growing single crystal waveguides in glass^18^, in addition, amorphous waveguides in glass^19^, depressed cladding^20^, type I^21^, and type II^22^ waveguides in bulk single crystal are all made possible by the spatially selective nonlinear absorption of fs laser irradiation in transparent materials. Crystals can be nucleated and grown because high repetition rates lead to heat accumulation^23^, which produces temperatures in and around the focal volume from hundreds to thousands of Kelvin^24^. The ability to produce ferroelectric crystals, such as lithium niobate (LiNbO_3_) and Ba_2_TiSi_2_O_8_ in glass, opens the opportunity to realize their large linear electro-optic effect and optical nonlinearity for use in electro-optic modulators and nonlinear frequency converters.

In particular, LiNbO_3_ is of special interest due to its favorable properties that have already found widespread applications in optical modulators, frequency converters, and acousto-optical filters^25^. LiNbO_3_ crystals in glass have previously been fabricated on both the surface of glass via CW laser irradiation^5^,^7^,^8^ and within glass via fs laser irradiation^16^,^17^. However, a significant problem remains that the fs laser precipitated LiNbO_3_ crystals in glass possess a noticeably non-uniform, polycrystalline structure^16^,^17^. These results point to a need to precisely control the interplay of nucleation and growth of crystals within the dynamic heating profile induced by the laser. It requires a careful optimization of fs laser parameters and glass composition. Taking into consideration the material constraints and desired application for the crystal, one could then select between various crystal growth modes such as an all-solid state glass → crystal transformation^26^ produced by a heat profile that yields a convex crystal growth front ahead of the scanning laser focus, or via melt → solid transformation^27^ that occurs with a concave crystal growth front behind the scanning laser focus.

In this report, we demonstrate the successful formation of uniform, highly oriented LiNbO_3_ crystals in lithium niobosilicate glass of effectively unlimited length, which is constrained only by the homogeneity of the starting glass. The specific growth dynamics and the confined nature of the crystal growth method lead to new phenomena in terms of crystal orientation^18^ that can be studied in our high quality, highly oriented single crystal line: (1) After nucleation, the crystal orientation rotates until its c-axis is oriented along the laser scanning direction. (2) Once the crystal is oriented in this way, we find a systematic, gradually varying misorientation of the crystal axis that is symmetric in regards to the center of the crystal line cross-section. The latter observation indicates that we have controlled the parameters such that the growth occurs upon heating in a convex growth front ahead of the scanning laser focus. We expand on the second characteristic of these crystal lines by detailing their potential application as graded index crystal waveguides in glass.

## Results

**Figure 1:**
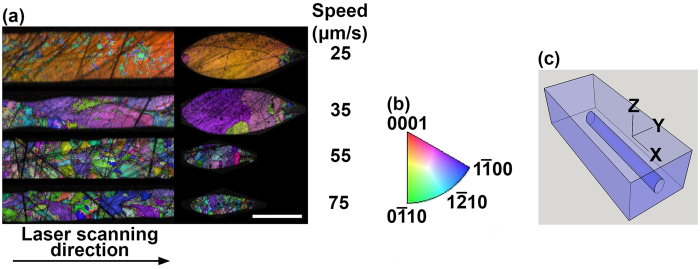
Effect of laser scanning speed on laser-induced formation of LiNbO_3_ crystals in glass. **(a)** Top and cross-section views of x-axis IPF maps for crystal lines written at a power density of 243 GW/m^2^ and at a scanning speed of 25, 35, 55, or 75 *μ*m/s. Scale bar corresponds to 25 *μ*m. **(b)** Inverse pole figure which assigns crystal directions to a certain color. **(c)** Coordinate system used throughout this work: the x-axis is parallel to laser scanning direction; the z-axis is perpendicular to sample surface; the y-axis is perpendicular to the x-axis and z-axis.

Figure 1(a) displays the influence of varying laser scanning speed on the crystal shape, size, and microstructure, while keeping the laser power density constant. The figure shows x-axis inverse pole figure (IPF) maps of crystal lines and their cross-sections for crystals written at a power density of 243 GW/m^2^ and at a scanning speed of 25, 35, 55, or 75 *μ*m/s. The crystals were grown in 3D deep inside the glass at a depth of 200 *μ*m below the surface. To prepare the sample for electron backscatter diffraction (EBSD) measurements, we brought the crystals to the glass surface by polishing. A schematic in Fig. 1(c) illustrates the coordinate system we used for EBSD analysis: the x-axis is parallel to the laser scanning direction; the z-axis is perpendicular to the sample surface; and the y-axis is perpendicular to both the x-axis and z-axis. Each IPF map is overlaid with an image quality map to darken areas where a quality Kikuchi pattern could not be measured, such as in the glass, at crystal grain boundaries, or along cracks or grooves created during polishing. Within the crystal, the Kikuchi patterns we observed correspond to a LiNbO_3_ crystal structure.

A trend is exhibited in Fig. 1(a) where the crystallized volume decreases with increasing scanning speed. In addition, the scanning speed has a clear impact on the degree of polycrystallinity of the crystal line as noted in terms of the number and size of grains of varying orientation (i.e. colors in the figure). We found parameters at which we could reliably create crystal lines where essentially the entire crystallized area in the cross-section is a single crystal (such as the 25 *μ*m/s case in Fig. 1(a)) with the c-axis nearly parallel to the scanning direction. As the scanning speed is increased, the single crystal is broken up into many grains, which increase in number and decrease in size.

**Figure 2:**
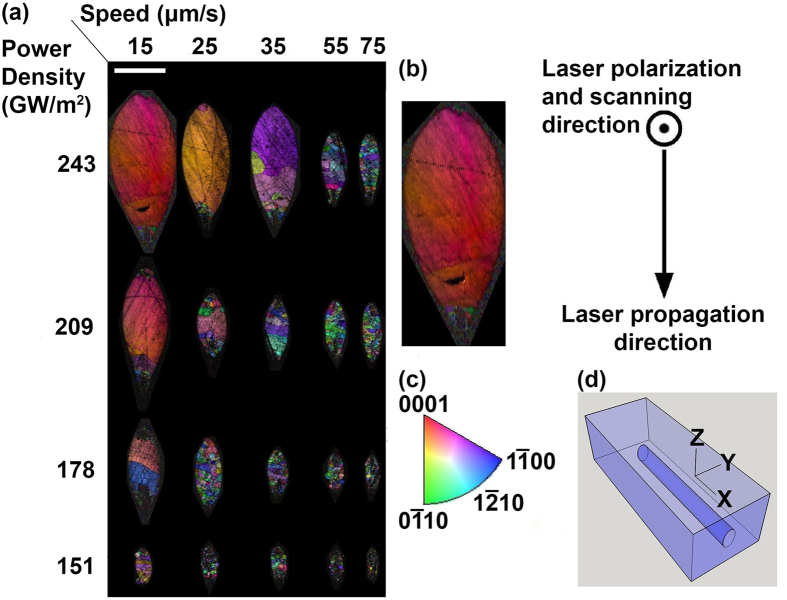
Effect of laser power density and scanning speed on laser-induced formation of LiNbO_3_ crystals in glass. (**a**) Cross-section view of x-axis IPF maps for crystal lines grown at a variety of laser scanning speeds and power densities. Scale bar corresponds to 25 *μ*m. (**b**) Close-up view of a crystal cross-section with laser parameters defined. (**c**) Inverse pole figure. (**d**) Coordinate system.

Expanding the search of the parameter space in terms of laser heating, Fig. 2(a) displays the combined influence of both laser scanning speed and power density on crystal growth. The figure shows x-axis IPF maps of the crystal line cross-sections for a variety of parameters. Several observations can be made from this figure. First, decreasing the power density reduces the crystallized volume, in agreement with the results of ref. 17. In addition, consistent with the results shown at 243 GW/m^2^ in Fig. 1(a), at all power densities, increasing the scanning speed results in a reduction of the crystallized volume. Also at all power densities, as the scanning speed is increased, there is a transition from single crystal cross-sections to polycrystalline cross-sections with decreasing grain size (20–30 *μ*m down to 2–3 *μ*m). It is clear from Fig. 2(a) that for lower power densities this transition occurs at slower speeds.

**Figure 3:**
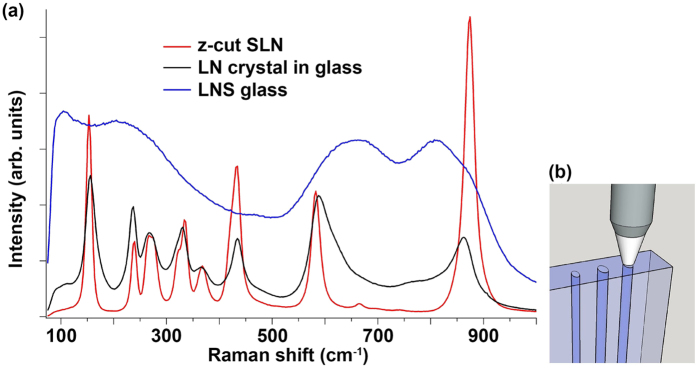
Raman spectroscopy of LiNbO_3_ crystal in glass. (**a**) Raman spectra acquired in a backscattering geometry for a c-axis oriented crystal line (grown at 209 GW/m^2^ and 15 *μ*m/s) and bulk z-cut stoichiometric LiNbO3. (**b**) Schematic of backscattering geometry.

We now focus our attention on the properties of the crystal lines formed with the best-case parameters (such as 243 GW/m^2^ and 15 *μ*m/s; 243 GW/m^2^ and 25 *μ*m/s; and 209 GW/m^2^ and 15 *μ*m/s). A Raman spectrum is shown in Fig. 3(a) for the crystal line grown at 209 GW/m^2^ and 15 *μ*m/s, along with Raman spectra for the host glass and a reference stoichiometric lithium niobate (SLN) bulk single crystal. Raman spectroscopy probes the vibrational modes of the crystal and reveals a collection of characteristic peaks, which allow us to identify the crystal structure. Consistent with the Kikuchi patterns acquired from EBSD, the Raman spectrum of the crystal in glass is indicative of a LiNbO_3_ crystal structure. The apparent peak broadening of the Raman modes of the crystal in glass relative to the stoichiometric, unconfined single crystal can be attributed to the crystal in glass deviating in composition from stoichiometric LiNbO_3_, as well as inhomogeneous stresses from confinement by the glass matrix.

In the backscattering geometry detailed in Fig. 3(b), one would expect that the crystal line was c-axis oriented, based on the x-axis IPF map of this crystal in Fig. 2(a). Then the Raman spectrum should appear as that of bulk z-cut LiNbO_3_ in backscattering geometry with laser probe beam incident along the c-axis. Our measurement of a bulk z-cut stoichiometric LiNbO_3_ Raman spectrum in Fig. 3(a) is indeed similar to the Raman spectrum of the crystal line grown in glass.

**Figure 4:**
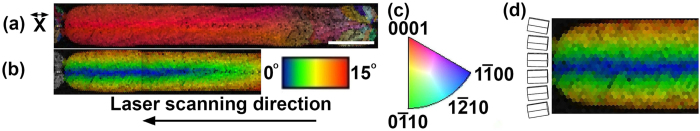
Lattice misorientation in laser induced crystal in glass. (**a**) X-axis IPF map of a crystal line grown at 209 GW/m^2^ and 15 *μ*m/s showing highly oriented growth over a 100 *μ*m length. Scale bar corresponds to 25 *μ*m. (**b**) Map of angular misorientation with respect to the c-axis oriented along the laser scanning direction. (**c**) Inverse pole figure. (**d**) Close-up view of the misorientation map at the left end of the crystal line along with lattices to illustrate the misorientation.

**Figure 5:**
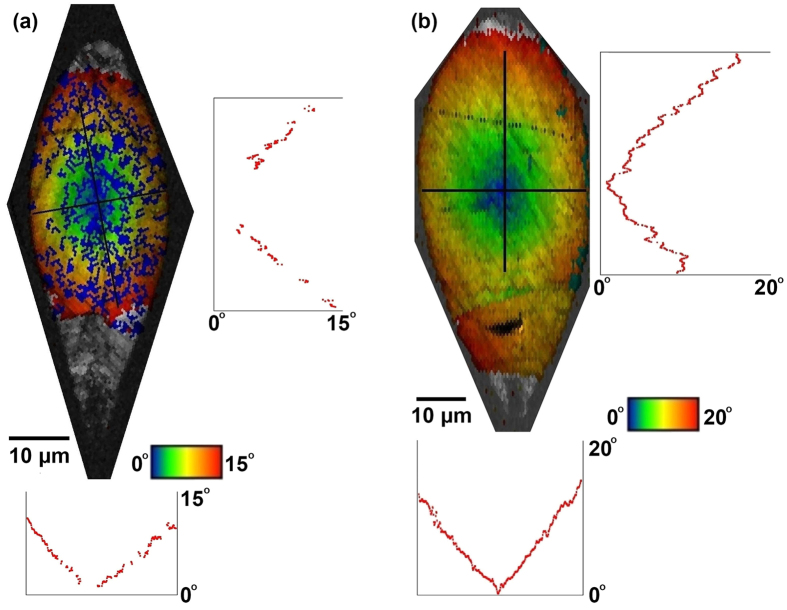
Line profiles of lattice misorientation in laser induced crystals in glass. Cross-section view of angular misorientation with respect to the c-axis orientation in the center of the crystal for crystal lines grown at (**a**) 209 GW/m^2^ and 15 *μ*m/s and (**b**) 243 GW/m^2^ and 15 *μ*m/s. The graphs below and to the right of each map are line profiles taken along the black lines on each map.

An x-axis IPF map of the crystal line written at 209 GW/m^2^ and 15 *μ*m/s in Fig. 4(a) exhibits uniform coloration that is indicative of single crystal over a length scale of 100 *μ*m. Also, Fig. 4(b) shows a map of angular misorientation with respect to the c-axis oriented parallel to the laser scanning direction, revealing a unique feature as yet unobserved in previous works. There is a discernible lattice misorientation which increases smoothly outward from the crystal center. EBSD provides us with the exact lattice orientation at each point in our map. However, a scalar misorientation map can lead to some ambiguity in the exact lattice orientation with respect to the reference direction. To clarify this, we show a representative case of the lattice orientation along the crystal line’s width in Fig. 4(d). Figure 5(a) features the angular misorientation in the crystal line’s cross-section, revealing a radially symmetric misorientation with respect to the center of the crystallized volume. The misorientation angle varies linearly as a function of distance from the center with a slope of 1.5°/*μ*m and reaches a maximum misorientation angle of 15°.

**Figure 6:**
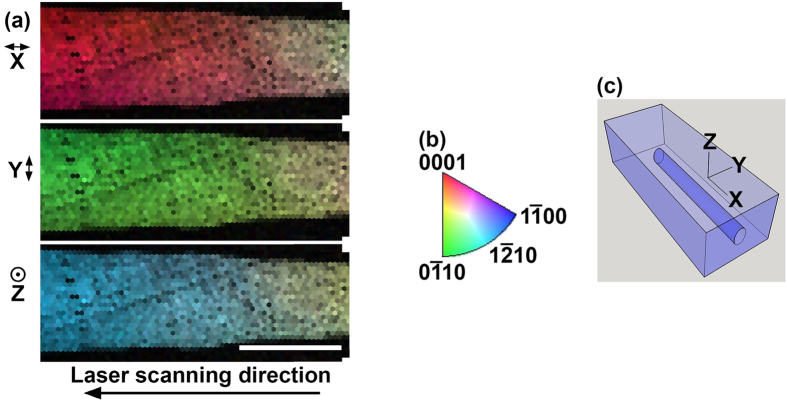
Lattice rotation towards c-axis orientation in laser induced crystal in glass. (**a**) IPF maps of a crystal line grown at 243 GW/m^2^ and 25 *μ*m/s showing tendency for lattice rotation to the c-axis parallel with the laser scanning direction. Scale bar corresponds to 25 *μ*m. (**b**) Inverse pole figure (**c**) Coordinate system.

Another intriguing aspect of the crystal growth is a gradual crystal rotation towards c-axis oriented crystal lines. This feature is somewhat evident in Fig. 1(a) for the crystal grown at 243 GW/m^2^ and 25 *μ*m/s. There, the x-axis IPF map shows a transition in color from greenish-orange to a deeper color orange as we go along the growth direction from left to right. A clearer example of such a rotation is shown in Fig. 6(a) for another crystal line grown under the same condition. The crystal ultimately aligns itself with the c-axis parallel to the laser scanning direction.

## Discussion

The results presented in Figs 1(a), 2(a) and 4(a) show that there are parameters of laser scanning speed and power density at which single crystals can be grown reproducibly. Very small polycrystalline structures appear at the top and bottom of these crystal cross sections. This likely results from a slight distortion of the temperature gradient at the top and bottom due to spherical aberration. The ability to grow crystals of practically unlimited length is an important step toward enabling us to evaluate the potential of LiNbO_3_ single crystal lines in glass for optical applications such as waveguiding.

We noticed an interesting feature where the crystal rotates until the c-axis is oriented parallel to the laser scanning direction. At the crystal’s nucleation point, such as at the right end of Fig. 4(a), a group of randomly oriented crystals are formed. A seed with its c-axis exactly parallel to the scanning direction is not at the leading edge or is not able to immediately dominate the growth. Rather, there is a gradual rotation from a random orientation toward the c-axis situated parallel to the laser scanning direction. Upon reaching this orientation the crystal continues to grow with c-axis orientation. Eventually, the growth is interrupted, as shown in Fig. 4(a), due to either glass inhomogeneity or translation stage instability. Subsequently, after a new nucleation occurs, the same process starts again. If this rotation can be controlled, we believe it may be useful in quasi-phase matching nonlinear interactions.

Some previous work can be brought into perspective by the present results. The correlation between laser scanning speed and crystal grain size presented in Figs 1(a) and 2(a) can explain why He *et al*.^17^ did not observe the formation of single crystal lines. Apparently, because the polycrystallinity begins to occur at slower speeds for lower power densities, even at the slow writing speed of 5 *μ*m/s used in that work, the low power density used would have resulted in polycrystalline structure, as predicted by the results of Fig. 2(a). Another aspect of their work, which can be explained, is the oscillation in brightness along the crystal lines in the second harmonic (SH) microscopy images. This is likely a result of the crystal rotation we observe in Figs 1(a) and 6(a). The x-axis IPF map in Fig. 6(a) shows that the crystal rotates to c-axis parallel to the laser scanning direction, and the y-axis IPF map shows that it was originally perpendicular to the laser scanning direction. Such a rotation would cause a significant variation in the SH microscopy images.

The lattice misorientation map in Fig. 4(d) gives us information about crystal alignment, from which we can infer details about the crystal growth dynamics. Other works have explained the tendency for oriented crystal growth along the laser induced temperature gradient^8^,^11^,^15^,^16^. We propose that the observed misorientation arises when crystal nucleation and growth occur during heating at the leading end of the laser focus. Nuclei formed at the leading end of the laser focus will align with their c-axis parallel to the temperature gradient, resulting in the lattice arrangement illustrated in Fig. 4(d). The result is a convex growth front where at each point along the front, the c-axis grows perpendicular to it.

It is remarkable that the misorientation continues all the way to the center of the crystal, as shown in Fig. 5(b). This could be a sign that the crystal is not being melted as the laser focus passes by. If melting had occurred, crystal nucleation and growth would reoccur upon cooling at the trailing end of the laser focus. Hence, if melting would have occured within the focus but off-center, the c-axis would tilt inward due to the orientation of the temperature gradient at the trailing end of the laser focus. However, we obeserve an outward tilt for the off-center area. The possibility that the very center of the crystal was melted cannot be completely ruled out since for that region the direction of the heat profile is identical during heating and cooling.

Fan *et al*.^16^ proposed that their fs laser written highly oriented crystals were the result of crystal growth during cooling, parallel to the temperature gradient, at the trailing end of the scanning laser focus. We have found parameters at which a different crystal growth mode occurs, similar to that reported recently by Savytskii *et al*.^26^ using a CW laser, resulting in crystallization during heating ahead of the laser focus. To our knowledge, we are the first to report such a crystal growth mode using a fs laser. Partly, this could be attributed to the lower repetition rate we used for our experiments (200 kHz instead of 300 kHz used in ref. 16). A lower repetition rate leads to less heat accumulation, directly impacting the temperature profile. The reduced temperature profile does not wipe out crystallization ahead of the laser focus, allowing this growth mode to dominate.

The temperature gradient resulting from nonlinear absorption of the laser beam dictates the crystal orientation, so the Gaussian distribution of the laser beam indeed impacts the crystal misorientation profile. Beam shaping via a spatial light modulator could allow for more complex temperature distributions, growth fronts, and crystal orientation profiles.

At present, we believe that the limiting factor which restricts the single crystal line length to ˜100*μ*m and ultimately causes a disruption in the crystal growth is glass inhomogeneity, appearing as striations within the glass. The stability of sample translation stage is also a likely source of disruption of crystal growth. Future work will include improvement of glass quality to reduce inhomogeneity, allowing for crystal growth to much longer dimensions.

Finally, we want to highlight the potential enhancement of the crystal line’s optical waveguiding capabilities due to the radial lattice misorientation observed in the crystal cross-section - see Fig. 5. As we will show in the following, the misorientation leads to a graded index crystal that enhances optical mode confinement. A graded index profile also offers other advantages such as a reduction in signal distortion due to modal dispersion and the ability to create a microlens^28^.

We deduce the refractive index profile within the crystals using the misorientation angle maps of Fig. 5. In a negative uniaxial crystal, such as LiNbO_3_, the refractive index experienced by the extraordinary ray varies with the angle *θ* between the propagation direction and the optic axis. The refractive index for the extraordinary ray is given by:


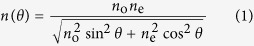


where *n*_e_ and *n*_o_ are the extraordinary and ordinary refractive indices, respectively^29^. The Taylor series expansion of equation (1) gives the following approximate expression for the refractive index profile:


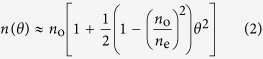


As shown in Fig. 5, the misorientation angle linearly increases with the radial distance from the center of the crystal. Thus, the refractive index profile has a parabolic spatial variation, the same as graded index fibers^28^:


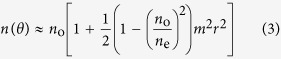


where m is the slope of the misorientation angle vs. position curve (about 1.5°/*μ*m) and r is the radial distance from the center of the crystal.

**Figure 7:**
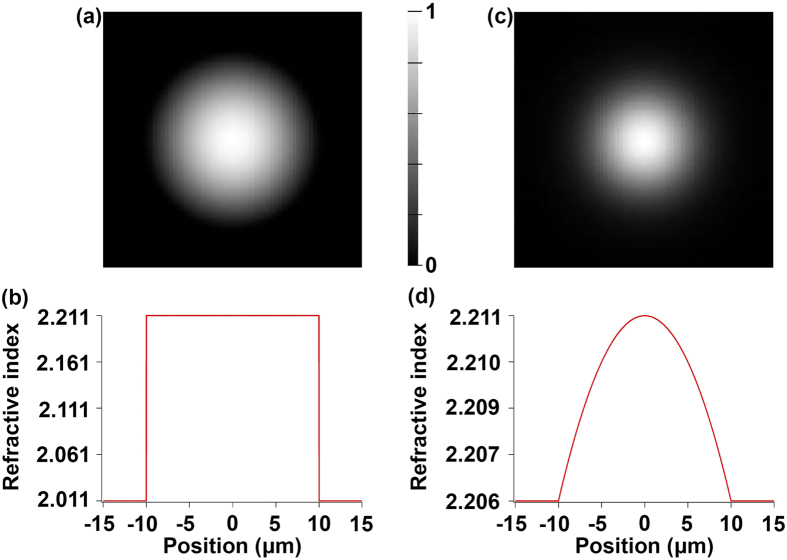
Optical waveguide modeling of laser induced crystals in glass. Fundamental quasi-transverse electric field mode profiles for 10 *μ*m radius (**a**) step index and (**c**) graded index waveguides. The corresponding (**b**) step and (**d**) graded refractive index profiles are shown beneath each mode profile. Note the different y-axis for each of the refractive index profiles.

One can compare the theoretical calculations of the mode profiles for radially symmetric graded index and step index waveguides. Applying equation (1), the misorientation of approximately 15° leads to a refractive index variation from the center to the edge of the crystal of approximately Δn_Gr._ = 0.0051, using the refractive indices of LiNbO_3_: *n*_e_ = 2.1381 and *n*_o_ = 2.2112 at *λ* = 1550 nm^30^. The step index between crystal and glass Δn_S.I._ was taken to be on the order of a few tenths: Δn_S.I._ = 0.2. Graded index and step index waveguides of 10 *μ*m radius were simulated using MODE solutions software. Each waveguide’s fundamental mode profile of the electric field, along with its corresponding refractive index profile, is shown in Fig. 7.

**Figure 8:**
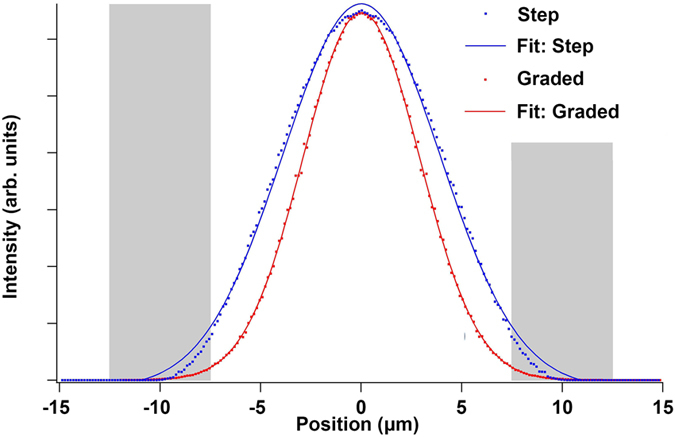
Comparison of different simulated waveguide intensity profiles at crystal-glass interface. Intensity line profiles of the fundamental modes for step index and graded index waveguides with their corresponding Gaussian fits. Gray areas highlight the crystal-glass interface.

All radial refractive index distributions can have their corresponding fundamental modes approximated by a Gaussian profile^31^. Shown in Fig. 8 is an intensity line profile for each waveguide along with its Gaussian fit. The full-width-at-half-the-maximum (FWHM) is 9.2 *μ*m for the step index mode and 6.8 *μ*m for the graded index mode. Most notable in terms of waveguiding properties, the graded index profile within the crystal dominates over the step index profile from crystal to glass. For the case of the graded index profile, the optical mode is almost completely guided within the crystal, reaching very little into the interface region between the crystal and the glass, shown schematically as gray blocks in Fig. 8. This mode confinement will drastically reduce the scattering at the rough crystal-glass interface. The advantage could be further enhanced by increasing the index gradient m. For example, a twofold increase of m from the current experimental values of 1.5°/*μ*m would further reduce the FWHM of the intensity profile to 4.8 *μ*m.

The crystals in glass grown in this work possess a step index from crystal to glass, and in addition, the crystals have been proposed to possess a graded refractive index. The large step index does not meet the weak waveguiding condition, a condition which dictates the degree of modal dispersion within the waveguide. The detriment of a waveguide failing to meet this condition lies in the fact that the modal dispersion would cause signal distortion during transmission.

However, the modal dispersion would not be impactful in the waveguide conceptualized in this work for two reasons. First, as is known for graded index glass fibers, the graded index profile of the waveguide we modeled would drastically reduce the modal dispersion. Second, these waveguides would not be used for long distance optical transmission, where time delays become significant.

In conclusion, an exploration of the parameter space of laser scanning speed and power density for growing LiNbO_3_ crystals within 35Li_2_O-35Nb_2_O_5_-30SiO_2_ glass led to virtually the entire crystallized volume as a single crystal. This accomplishment eliminates the problem of polycrystallinity in a large percentage of the crystallized volume which would be detrimental to the potential waveguiding capabilities of the crystal line. We observe a propensity for the crystal line’s lattice to rotate during growth until the c-axis is oriented along the laser scanning direction. A growth mode where nucleation and growth of the crystal line occur upon heating and ahead of the laser focus was established by optimally adjusting the parameters of laser power density and scanning speed. This growth mode was confirmed by the radially symmetric crystal lattice misorientation with respect to the center of the crystal. We contend that the resultant graded index profile due to this lattice misorientation would improve the crystal line’s waveguiding ability through an enhanced mode confinement, reducing scattering at the crystal-glass interface.

## Methods

### Glass preparation

Lithium niobosilicate glass of composition 35Li_2_O-35Nb_2_O_5_-30SiO_2_ was made by mixing high purity SiO_2_ (99.99%), Li_2_CO_3_ (99.999%), and Nb_2_O_5_ (99.9985%) reagents into a 40 g batch. The mixture was melted in a Pt crucible at 1400 °C for one hour, and the melt was subsequently quenched between two steel plates at room temperature. The glass was annealed in an oven at 500 °C for 2 hours to relieve stresses, before cooling down slowly to room temperature. The glass was then cut into rectangular pieces of roughly 10 mm × 10 mm × 2.5 mm dimension and polished to optical quality.

### Laser-induced crystallization

A PHAROS femtosecond laser (model: SP-06-200-PP, Light Conversion, Vilnius, Lithuania) was used to create the crystals in glass. The laser was operated at a wavelength of 1026 nm, repetition rate of 200 kHz, and pulse duration of 175 fs. The laser polarization was oriented parallel to the laser scanning direction. The light was focused through a Nikon extra-long working distance 50x objective with 0.6 NA. The glass piece was mounted on a heated stage brought to 500 °C to eliminate cracking during crystal growth. The heated stage was mounted on motorized stages, enabling 3D motion of the laser focus within the glass. Laser scanning speed was varied between 15 and 75 *μ*m/s and laser power density (power) was varied between 151 GW/m^2^ (515 mW) and 243 GW/m^2^ (830 mW). The power was measured after the objective, but before the heated stage’s 1 mm thick silica window. The crystals were grown at an actual depth of 200 *μ*m below the surface of the glass.

### Materials characterization

After writing crystal lines at the specified parameters, EBSD measurements and analysis were carried out using a Hitachi 4300SE scanning electron microscope and the analysis package Orientation Imaging Microscopy Analysis, respectively. Raman measurements were made with a WITec Raman microscope (alpha300 RA) utilizing a 532 nm laser. The samples for these analyses were either cut and polished to expose the crystal cross-sections or polished down from the top surface to expose the crystals along their length. In either case, the sample was polished with progressively finer grits down to a 0.1 *μ*m finish. To simulate the dependence of the waveguiding characteristics on the corresponding refractive index profile, we utilized the MODE Solutions software (Lumerical Solutions, Inc.). To simplify the calculation, the waveguides were reduced to cylindrical symmetry.

## Additional Information

**How to cite this article:** Veenhuizen, K. *et al*. Fabrication of graded index single crystal in glass. *Sci. Rep.* **7**, 44327; doi: 10.1038/srep44327 (2017).

**Publisher's note:** Springer Nature remains neutral with regard to jurisdictional claims in published maps and institutional affiliations.
